# Unlocking the potential of wearable technology: Fitbit-derived measures for predicting ADHD in adolescents

**DOI:** 10.3389/frcha.2025.1504323

**Published:** 2025-05-22

**Authors:** Muhammad Mahbubur Rahman

**Affiliations:** ^1^ Center for Translational Research, Children’s National Hospital, Silver Spring, MD, United States; ^2^ Pediatrics & Biostatistics and Bioinformatics, George Washington University, Washington, DC, United States

**Keywords:** ADHD, fitbit-derived physical activity, wearable technology, adolescent mental health, machine learning

## Abstract

**Background:**

Attention-deficit/hyperactivity disorder (ADHD) is a common neurodevelopmental disorder with a complex etiology. The current diagnostic process for ADHD is often time-intensive and subjective. Recent advancements in machine learning offer new opportunities to improve ADHD diagnosis using diverse data sources. This study explores the potential of Fitbit-derived physical activity data to enhance ADHD diagnosis.

**Method:**

We analyzed a sample of 450 participants from the Adolescent Brain Cognitive Development (ABCD) study (data release 5.0). Correlation analyses were conducted to examine associations between ADHD diagnosis and Fitbit-derived measurements, including sedentary time, resting heart rate, and energy expenditure. We then used multivariable logistic regression models to evaluate the predictive power of these measurements for ADHD diagnosis. Additionally, machine learning classifiers were trained to automatically classify individuals into ADHD+ and ADHD− groups.

**Results:**

Our correlation analyses revealed statistically significant associations between ADHD diagnosis and Fitbit-derived physical activity data. The multivariable logistic regression models identified specific Fitbit measurements that significantly predicted ADHD diagnosis. Among the machine learning classifiers, the Random Forest outperformed others with cross-validation accuracy of 0.89, AUC of 0.95, precision of 0.88, recall of 0.90, F1-score of 0.89, and test accuracy of 0.88.

**Conclusion:**

Fitbit-derived measurements show promise for predicting ADHD diagnosis, with machine learning classifiers, particularly Random Forest, demonstrating high predictive accuracy. These findings suggest that wearable data may contribute to more objective and efficient methods for ADHD identification, potentially enhancing clinical practices for diagnosis and management.

## 1 Introduction

Attention-deficit/hyperactivity disorder (ADHD) is a common neurodevelopmental disorder in childhood and may persist into adulthood, affecting about 9.8% of U.S. children (1) and 4.4% of adults (2). During childhood, it often presents with inattention, hyperactivity, and impulsivity, such as difficulty focusing on tasks, excessive movement, and acting without consideration (3). These symptoms often result in significant challenges, disrupting academic performance (e.g., incomplete schoolwork), social interactions (e.g., strained peer relationships), and behavioral regulation (e.g., difficulty following rules). As individuals transition into adulthood, these symptoms shift, with hyperactivity often lessening, while persistent inattention and impulsivity are commonly paired with challenges like emotional dysregulation and impaired executive functioning (4). ADHD often co-occurs with anxiety (1, 5) and depression (6), highlighting its complexity. Misdiagnosis can lead to substance abuse, lower education attainment, and legal issues (7–9). However, diagnosing ADHD is hindered by barriers like limited understanding, time-consuming assessments, and subjectivity (10, 11). The co-occurrence of similar conditions adds to the challenge (12). Machine learning approaches can leverage valuable evidential information in automatic ADHD diagnosis.

Many studies have applied machine learning to predict ADHD, using various data sources such as continuous performance test (CPT) variables (13), pupillometric biomarkers and time series (14), EEG measurements (15), brain signals (16), brain connectome topological information (17), functional MRI (18), symptom ratings and neuropsychological measures (19), 3D MR images (20), and fMRI from ABCD study (21).

Research suggests a complex relationship between physical activity, sedentary behavior, and ADHD. For instance, some studies have reported increased physical activity levels in children with ADHD (22), while others have linked higher resting heart rate (RHR) and lower step counts to greater internalizing symptoms (23). Additionally, associations between physical activity and mental health symptoms (24, 25), as well as the negative impact of sedentary behavior on mental health (26) have been documented. Physical activity has also been associated with improved executive function, a cognitive domain often impaired in individuals with ADHD (27).

Evidence from self-reported data indicates a link between ADHD symptoms and sedentary behavior (28). Interestingly, sedentary activities like reading and studying have been found to enhance executive function and academic skills, suggesting that not all sedentary behaviors are detrimental (29). Heart rate related studies have further demonstrated higher heart rates in children with ADHD (30), with similar findings observed in adults with ADHD, particularly those on stimulant medication (31, 32).

In terms of energy expenditure, stimulant medications for ADHD have been associated with reduced daily energy expenditure in children (33). However, individuals with ADHD tend to have higher resting energy expenditure (34). Particularly, greater energy expenditure in late adolescence has been linked to lower ADHD scores (35).

In most machine learning studies for ADHD diagnosis, researchers primarily relied on either brain images or EHR collected in lab or hospital settings. This approach, while informative, poses several challenges, including high costs, time-intensive, and ethical concerns regarding the potential inclusion of sensitive personal information when training machine learning models. Additionally, many investigations exploring the relationship between various physical activities and ADHD faced limitations stemming from small sample sizes, potentially compromising the representativeness and generalizability of their findings. Moreover, a substantial portion of these studies relied on self-reported data, which introduced the risk of recall bias and inaccuracies, potentially failing to capture the full spectrum of sedentary time and energy expenditure, thereby impacting result precision. Furthermore, the majority of the data were collected from a single site, which could limit the broader applicability of the analysis.

Nonetheless, the collection of physical activity summaries could be significantly enhanced by leveraging smartphone sensors and wearable devices such as Fitbit and smart watch. Our study aimed to address these challenges by harnessing data from the Adolescent Brain Cognitive Development (ABCD) study, an extensive, long-term study encompassing 11,874 adolescents across 21 research sites in the United States. This dataset includes comprehensive Fitbit measurements, providing interesting daily and weekly physical activity summaries that cloud offer invaluable insights into sedentary time, RHR, and energy expenditure investigation for a significant number of adolescents, both with and without ADHD. The primary goal of our study was to investigate potential correlations between Fitbit measurements, including sedentary time, RHR, and energy expenditure, and ADHD diagnosis, as well as their predictive capabilities in ADHD diagnosis through the development of machine learning models.

Our primary contributions are as follows:

• Establishing associations between Fitbit measurements, including sedentary time, RHR, and energy expenditure, and ADHD diagnosis.
• Investigating the complex relationship between ADHD diagnosis and various independent Fitbit measurements.
• Developing predictive models for ADHD in adolescents using Fitbit measurements and conducting a comprehensive comparative analysis across multiple machine learning algorithms.
• Leveraging the extensive ABCD dataset to better understand ADHD diagnosis by analyzing daily and weekly physical activity summaries collected via wearable devices like Fitbit.

**Figure 1:**
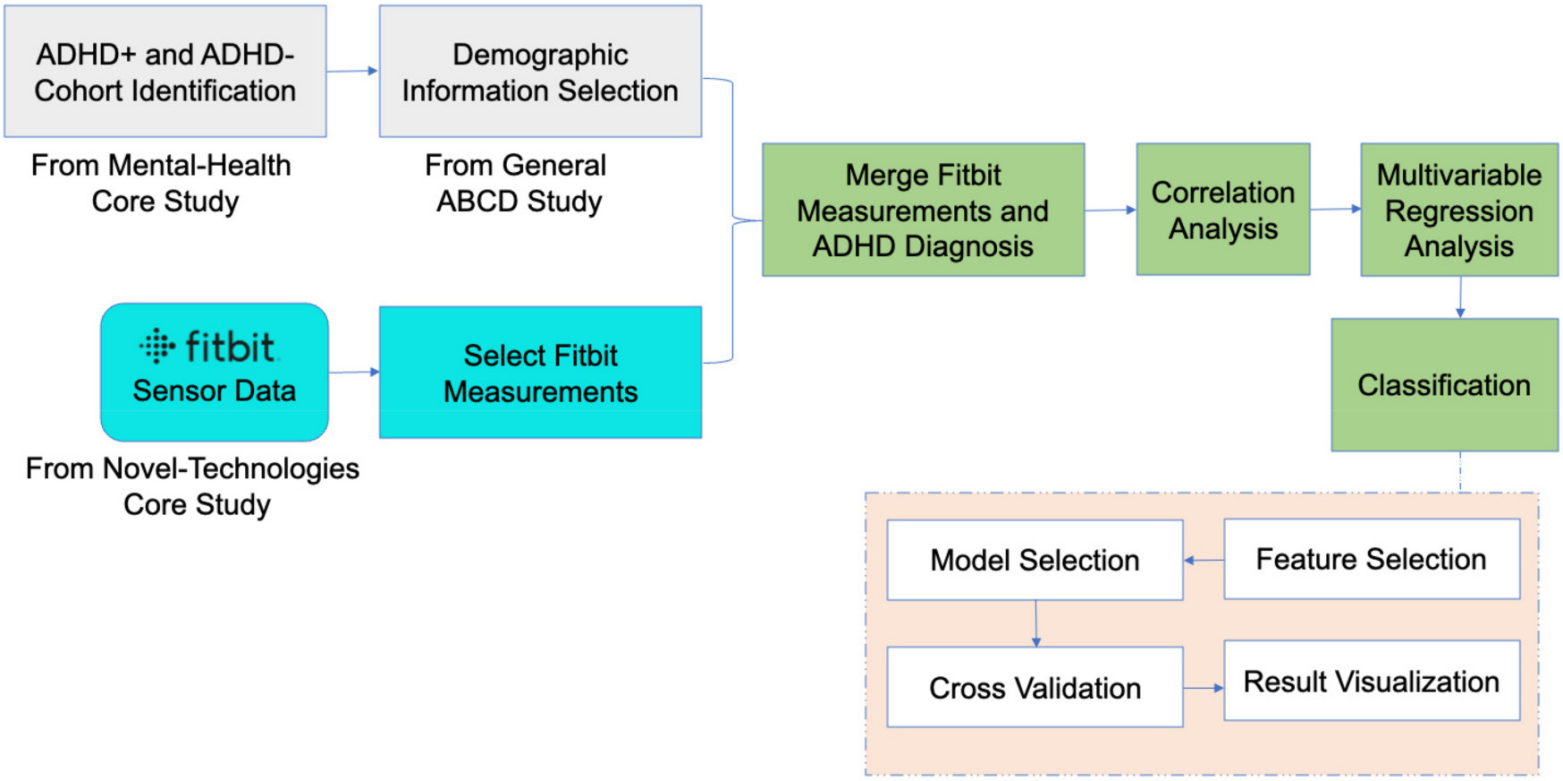
Overview of the study design and methodology, including cohort identification, data integration, analysis procedures, and model selection. The figure illustrates the key stages of the research: (1) identification of ADHD+ and ADHD− groups from the ABCD dataset based on diagnostic criteria and inclusion/exclusion rules; (2) integration of Fitbit data for daily and weekly activity summaries; (3) statistical analysis to explore relationships between ADHD status and Fitbit measurements; (4) predictive modeling using various machine learning algorithms to predict ADHD diagnosis.

To the best of our knowledge, the integrated investigation of these Fitbit measurements from ABCD study to predict ADHD diagnosis represents a novel approach that has not been previously explored. Additionally, our modeling incorporates demographic information of adolescents, influenced by a study conducted by Nagata et al. within the ABCD framework, which demonstrated associations between sociodemographic variables and physical activities, such as step counts using Fitbit (36). Figure 1 shows the overview of our whole study.

## 2 Materials and methods

### 2.1 Study participants

In this study, the data were obtained from the ABCD research consortium. The ABCD study enrolled a total of 11,874 children, aged between 9 and 10, from 21 different study sites across the United States. For the purpose of our research, we used the ABCD Parent Diagnostic Interview for DSM-5 Full (KSADS-5) sub-study. In data release 5.0, the criteria for diagnosing ADHD were modified to necessitate impairment in two domains, as opposed to the earlier release that relied on impairment in only one domain. This sub-study enabled us to specifically identify subjects with ADHD positive (ADHD+) and ADHD negative (ADHD−) for our research cohort.

#### 2.1.1 ADHD+ group

Within the cohort of adolescents with ADHD, we included individuals who had a diagnosed ADHD condition at the time or who were in partial remission from ADHD. Additionally, we excluded any individuals who were diagnosed with ADHD in the past for a minimum of one school year but no longer exhibit ADHD symptoms (i.e., subjects who were fully in remission from ADHD). As detailed in the ABCD study's data release 5.0, the determination of an ADHD diagnosis was calculated by evaluating impairment across at least two domains (e.g., the ability to engage in goal-directed behavior and the capacity to refrain from impulsive actions) (37). A total of 357 individuals were identified as ADHD+ based on the inclusion and exclusion criteria.

#### 2.1.2 ADHD− group

For the selection of ADHD− participants, we included adolescents who had never been diagnosed ADHD. Furthermore, we ensured that this group did not include individuals who were either partially or fully in remission from ADHD, nor those who were diagnosed with ADHD during any school year throughout their lifetime. However, we did not take into account the presence of any other mental health conditions when defining this ADHD− cohort. A total of 3311 unique individuals were identified as ADHD− based on the inclusion and exclusion criteria.

### 2.2 Fitbit measures

The ABCD Youth Fitbit daily physical activity summaries (*n* = 7,439) involved the assessment of daily physical activity and sedentary behavior at the minute level, utilizing heart rate and accelerometer data from Fitbit sensors worn by adolescents. Additionally, the ABCD Youth Fitbit weekly physical activity summaries (*n* = 7,076) captured weekly physical activity and sedentary behavior, including only days with adequate wear time for inclusion (>600 min of daytime wear) from the Fitbit sensors worn by adolescents. These datasets encompassed minutes spent in various activity intensities and recorded step counts, categorized into weekdays, weekends, daytime, nighttime, and all days of the week. Fitbit data was collected at baseline, the 2-year follow-up, and the 4-year follow-up using the Fitbit Charge 2 model worn on the wrist with parental consent. The participants wore Fitbit consistently for a period of over 21 days except during bathing and any water activities. Our study integrated Fitbit measurements across all three phases with minimal participants overlap. Data from both activity summaries were used in our Fitbit measurements of participant's daily and weekly physical activity summaries, providing essential measurements relevant to our research goal. The specific measurements utilized in our study are shown in Table 1, fall within three primary categories: sedentary time, resting heart rate, and energy expenditure. The measurement definitions were directly taken from ABCD data.

**Table 1:** Fitbit measurements used in the study to assess physical activity in relation to ADHD diagnosis.

Measurement	Category	Description (According to ABCD data)
fit_ss_total_sedentary_min	Sedentary time	The total number of minutes of sedentary (<1.5 METS) time observed over a 24-h period, from midnight (00:00) to 11:59 PM (23:59), including periods of inactivity during sleep.
fit_ss_fitbit_sedentarymin		The total number of minutes spent in sedentary (<1.5 METS) time during the day.
fit_ss_dayt_sedentary_min (non-sleep)		Number of minutes of sedentary (<1.5 METS) time observed during non-sleep (night) valid minutes.
fit_ss_wk_avg_sedentary_min		Weekly average minutes spent in sedentary (<1.5 METS) during day.
fit_ss_fitbit_rest_hr	Resting heart rate (RHR)	Weekly average resting heart rate during day.
fit_ss_fitbit_restingheartrate		Daily avg resting heart rate for the day from daily level summary.
fit_ss_wk_average_met_value	Energy expenditure	Weekly average METS/min during day on included days.
fit_ss_dayt_ave_met_value		Daily average METS/minute during non-sleep (night) valid minutes.
fit_ss_total_ave_met		Average METS/minute of all valid minutes from midnight (00:00) to 11:59 PM (23:59) regardless of sleep status.

The key variables from the Fitbit data include daily and weekly summaries of sedentary time, resting heart rate, and energy expenditure. These measurements were categorized into three main groups—sedentary time, resting heart rate, and energy expenditure—to examine their association with ADHD status (ADHD+ vs. ADHD−).

“Sedentary time” encompasses extended periods of inactivity or limited physical activity, signifying the duration spent in a seated, reclined, or lying position with minimal bodily movement and low energy expenditure. It commonly includes activities such as sitting at a desk, watching television, or using a mobile phone, where individuals engage in little to no physical effort. “Resting heart rate” represents the number of heart beats per minute during periods of rest. This measurement is typically taken while an individual is awake, in a state of relaxation, and not involved in any physical activity. Monitoring RHR serves as a useful tool for assessing overall well-being and tracking changes in fitness over time. “Energy expenditure,” expressed as METs/min (metabolic equivalents per minute), is a metric used to gauge the rate of energy consumption during various activities. An MET minute represents the energy expended within a minute of activity while at rest. To put it simply, 1 MET corresponds to the resting metabolic rate of an average adult, estimated at around 3.5 milliliters per kilogram per minute of oxygen consumption (ml/kg/min).

We conducted thorough data cleaning and preprocessing, eliminating rows with entirely missing values. If a single cell value was missing for a particular subject, we employed data imputation by replacing the missing data with the mean value of that specific subject's variable. The detailed data processing steps are given in the Supplementary Material: data processing and merging section.

### 2.3 Study data sample

The Fitbit data was integrated with the ADHD+ samples, resulting in the identification of Fitbit data for specific measurements/variables in 225 out of the 352 ADHD+ subjects. Consequently, our final group of ADHD+ participants consisted of 225 distinct subjects. Likewise, among the 3,311 ADHD− subjects, 2,230 unique participants had corresponding Fitbit data, forming our ADHD− sample of 2,230 distinct subjects.

Given that our ADHD+ subject count reached 225, an effort was made to balance the participant list by selecting nearly equivalent ADHD− subjects from a pool of 2,230 candidates. This selection was performed through stratified sampling, considering factors like gender, age, race, parent's education, marital status, and income level (Supplementary Table S1). This approach ensured a harmonized distribution between ADHD+ and ADHD− samples.

Ultimately, our dataset comprises 450 distinct adolescents, evenly split between ADHD+ and ADHD− subjects. The dataset is organized in a long format, featuring repeated observations for each subject over 21 days across different variables. It includes 10,045 Fitbit data records for 450 participants (ADHD+ and ADHD−), with a few having data beyond 21 days. Details on data merging, including explanations for retaining additional days for some participants, are available in the Supplementary Material: data processing and merging section. In the data release 5, a large difference between the fit_ss_fitbit_sedentarymin measurement and other sedentary min variables may indicate poor data capture at the minute level. However, it is important to note that fit_ss_fitbit_sedentarymin reflects only daytime activity, while other sedentary minute variables measure activity over the entire day (24 h), night, or week. As a result, directly calculating differences among these variables using the released tabular data can be challenging. Therefore, to minimize the impact of extreme values or outliers, we applied data normalization techniques, scaling the variables to a consistent range. This approach allowed mitigating the risk of potential false positives.

### 2.4 Data analysis

In our study, statistical and predictive analyses were conducted. The statistical analysis was primarily carried out using correlation analysis. Additionally, confirmatory analysis, involving variable selection for machine learning models, was performed using multivariable logistic regression analysis. Subsequently, a predictive analysis was conducted using machine learning methods that included classification algorithms.

#### 2.4.1 Statistical analysis

Initially, a Pearson correlation analysis was conducted to examine the linear association between ADHD diagnosis (i.e., ADHD+ and ADHD− groups) and various Fitbit measurements. These Fitbit measurements included participants' various energy expenditures, RHR, and sedentary time during different time intervals (Table 1). This correlation analysis was performed between-subjects comparisons, both with and without age, gender, race, parent's education, and income level as control. A separate analysis using only age as a covariate was also conducted to observe its effects. For the between-subjects model, the independent variables included demographic characteristics (e.g., age, gender, race, and parent's education) as well as Fitbit measurements. The dependent variable was the ADHD diagnosis. Control variables were adjusted for in the analysis to account for potential confounding effects.

Additionally, a repeated measures correlation analysis was conducted to explore within-subjects associations. For the within-subjects analysis, the independent variables remained the same (demographic characteristics and Fitbit measurements). The dependent variable was again the ADHD diagnosis. This model accounted for repeated measures from the same individuals across different time points, allowing for the assessment of within-subject variability and more accurate estimation of the relationships over time. To address potential false discoveries, a *p*-value correction using Holm's Sequential Bonferroni method (38) was applied. Corrections were applied to both between- and within-subjects analyses.

Subsequent to the initial correlation analysis, a multivariable logistic regression analysis was conducted using the Maximum Likelihood Estimation (MLE) method. This approach was chosen due to our focus on predicting binary outcomes at the participant level, where daily observations are considered independent. The logistic regression model was trained on 80% of the whole dataset, with the remaining 20% reserved for evaluating the machine learning models. This analysis aimed to investigate the relationship between ADHD diagnosis and independent Fitbit measurements. However, in this analysis, the repeated measures were handled through the inclusion of time (date) as covariate in modeling. The results of this analysis informed the selection of variables for our machine learning model, designed to predict binary ADHD diagnosis outcomes. Additionally, we conducted mixed effects modeling to assess subject-specific variability. The mixed-effects model included time and various Fitbit measurements as independent variables, with ADHD diagnosis as the dependent variable, treated as a binary outcome. To account for individual variability in the diagnosis across repeated measures, a random intercept for participants (subject_id) was incorporated. The model employed a generalized linear mixed-effects framework with a binomial distribution and utilized the BOBYQA optimizer to ensure robust estimation and convergence. Collinearity was evaluated by calculating the Variance Inflation Factor (VIF) and managed separately by scaling the variables and employing Principal Component Analysis (PCA).

#### 2.4.2 Predictive analysis and classification

A range of supervised machine learning algorithms was implemented to predict ADHD diagnosis, with a focus on distinguishing between ADHD+ and ADHD− subjects. These algorithms included Decision Tree (DT), Random Forest (RF), Naïve Bayes (NB), AdaBoost (Ada) classifier, Light Gradient Boosting Machine (LGBM) classifier, Logistic Regression (LR) classifier, Support Vector Machines (SVM) classifier with non-linear kernels, and K Nearest Neighbors (KNN) classifier. To optimize the classification models, a grid search with 10-fold cross-validation was conducted on the 80% of the data, and a multi-core implementation was utilized to fine-tune the hyperparameters. Upon the identification of the best hyperparameters, the models were trained using these settings. To reduce the risk of overfitting in DT and RF, we applied diverse strategies such as pruning, optimizing the number of samples per leaf, and increasing the number of trees.

Our models were trained using Fitbit measurements that demonstrated statistical significance according to our multivariable logistic regression analysis, in addition to the demographic variables such as participants' age, gender, race, parent's socioeconomic status, and parent's education. To prepare for training, essential preprocessing steps were taken, encompassing the removal of duplicates, conversion of categorical variables into numerical representations, and data normalization. These steps were essential to ensure compatibility with the classification algorithms.

The models were evaluated using a range of performance metrics. In the training process for all models, 10-fold cross-validation was applied on the 80% of the training data. Furthermore, separate models were trained on 80% of the data, and its performance was evaluated on the remaining unseen 20% of the dataset. Accuracy, precision, recall, and F1-scores were computed using both the 10-fold cross-validation and test data. Additionally, learning curves and ROC curves, along with AUC scores, were generated to assess the models' ability to generalize.

Python 3.9 and R version 4.3.2 were utilized for all analyses. Data processing was carried out with the Pandas and Numpy libraries. Correlation analyses were performed using the Statsmodels libraries. Multivariable logistic regression and mixed effects regression analyses were conducted using lme4 and car packages. The development of machine learning models was done using the Scikit-Learn and LightGBM machine learning libraries, with multi-core processing to optimize efficiency. Data and results visualizations were created using the Matplotlib, Seaborn and Pandas packages.

## 3 Results

The study had 450 participants whose demographic details are presented in Table 2. Supplementary Table S3 provides demographic characteristics for the ADHD+ group, while Supplementary Table S4 presents corresponding information for the ADHD− (control) group.

**Table 2:** Demographic characteristics of study participants.

Description	M	SD
*n* *=* *450*		
Age		
Overall	9.45	0.50
	*n*	%
Gender		
Male	257	57.11
Female	191	42.44
Other	2	0.44
Race		
White	368	81.77
African American	60	13.33
Chinese	3	0.66
American Indian	2	0.44
Asian Indian	1	0.22
Other	16	3.55
Ethnicity		
Not Hispanic	368	81.77
Hispanic	73	16.22
Refused to answer	2	0.44
Don't know	7	1.55
Parent's Education		
Bachelor's degree	128	28.44
Master's degree	119	26.44
Some college	70	15.55
Associate degree	57	12.66
High school	39	8.66
Professional degree	14	3.11
Doctoral degree	15	3.33
GED or equivalent Diploma	6	1.33
Refused to answer	2	0.44
Parent's Income Level		
$100,000 to $199,999	139	30.88
$50,000 to $99,999	121	26.88
$200,000 and greater	57	12.66
$25,000 to $49,999	54	12.00
Less than $25,000	44	9.77
Refuse to answer	20	4.44
Don't know	15	3.33

M, mean; SD, standard deviation.

*n* is the number of participants.

**Figure 2:**
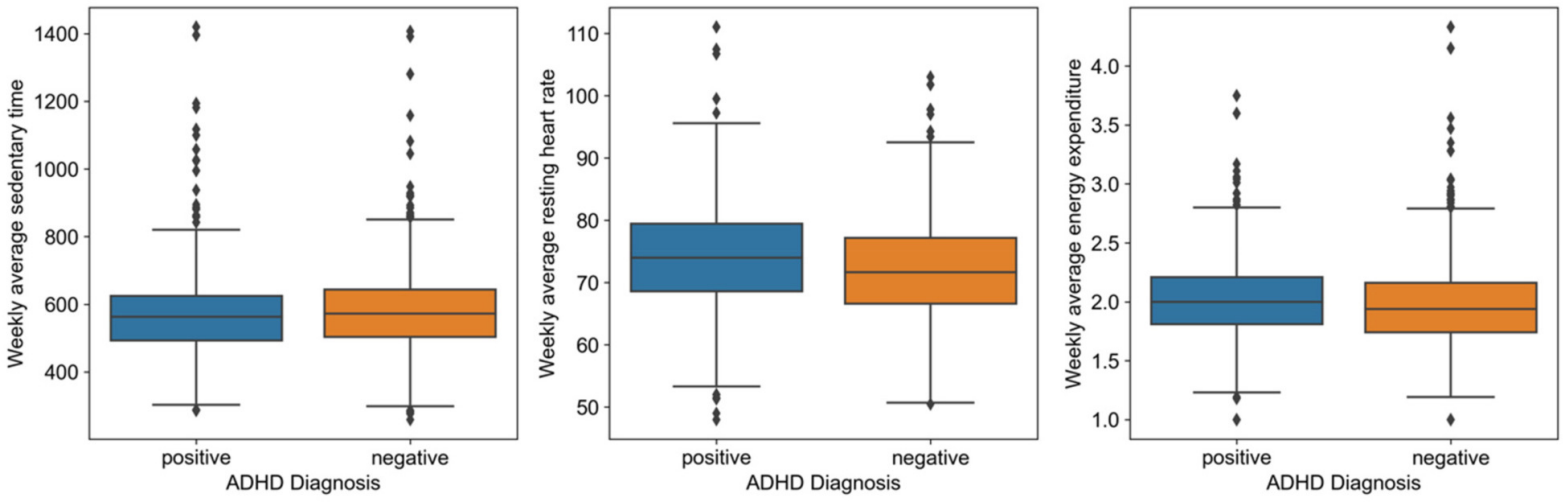
Variability in weekly Fitbit measurements between ADHD+ and ADHD− groups. Box plots showing weekly average sedentary time (in minutes), resting heart rate (in beats per minute), and energy expenditure (in METS/min) for each group. The *x*-axis indicates the ADHD diagnosis groups (ADHD+ vs. ADHD−) and the *y*-axis represents the measurement values. The blue box is ADHD+ group, and the orange box is ADHD− group.

Figure 2 shows measurements variability for weekly average sedentary time, RHR and energy expenditure with ADHD diagnosis. Based on the box plots and the presence of more data points above the maximum line in ADHD+ for RHR, there was indeed more variability in these Fitbit-derived measures among individuals with ADHD+ compared to ADHD−. To further investigate, we conducted descriptive statistics and the Fligner-Killeen test to assess variability in sedentary time, RHR, and energy expenditure between the ADHD+ and ADHD− groups. The analysis revealed greater variability in RHR and sedentary time within the ADHD+ group. Descriptive statistics showed that the ADHD+ group had a higher mean RHR (74.02 bpm) than the ADHD− group (72.01 bpm), with greater dispersion (std = 11.99 vs. 10.52). Sedentary time also exhibited slightly higher variability in the ADHD+ group (std = 117.93) compared to the ADHD− group (std = 111.89). In contrast, energy expenditure demonstrated minimal variability between the groups. The Fligner-Killeen test confirmed these findings, with significant differences in variance for RHR (Fligner Statistic = 15.14, *p* = 0.0001) and sedentary time (Fligner Statistic = 4.36, *p* = 0.037), indicating higher variability in the ADHD+ group. No significant variance differences were found for energy expenditure (Fligner Statistic = 0.07, *p* = 0.78). Detailed test results were given in Supplementary Tables S7 and S9.

### 3.1 Association between diagnosis and measurements

During the Pearson correlation analysis conducted between ADHD diagnosis and Fitbit measurements without controls, statistically significant findings were obtained for between-participant analysis. However, the observed correlation coefficients were relatively small in magnitude. Statistically significant correlations were observed in most cases when demographics were used as controls. Notably, within-participant analyses demonstrated significance in specific scenarios shown in Supplementary Table S2. Additionally, the results were also significant when age was employed as the sole covariate (Supplementary Table S2). Table 3 provides the correlation analysis between ADHD diagnosis and Fitbit measurements. All reported *p*-values are post-correction using Holm's Sequential Bonferroni method. Corrections were applied separately for multiple tests within the groups of between-participants, within-participants, and with-controls analyses.

**Table 3:** Correlation analysis between ADHD diagnosis and Fitbit measurements.

Fitbit measurements	Between participants	
	Without controls	With controls
fit_ss_dayt_sedentary_min (non-sleep)	*r*: −0.041	*r*: −0.025
	*p* < 0.0001	*p* = 0.013
fit_ss_fitbit_sedentarymin	*r*: 0.070	*r*: 0.060
	*p* < 0.0001	*p* < 0.0001
fit_ss_wk_avg_sedentary_min	*r*: −0.038	*r*: −0.016
	*p* < 0.0001	*p* = 0.119
fit_ss_total_sedentary_min	*r*: −0.081	*r*: −0.058
	*p* < 0.0001	*p* < 0.0001
fit_ss_fitbit_rest_hr	*r*: 0.122	*r*: 0.161
	*p* < 0.0001	*p* < 0.0001
fit_ss_fitbit_restingheartrate (day only)	*r*: 0.119	*r*: 0.160
	*p* < 0.0001	*p* < 0.0001
fit_ss_dayt_ave_met_value	*r*: 0.041	*r*: 0.003
	*p* < 0.0001	*p* = 0.769
fit_ss_wk_average_met_value	*r*: 0.058	*r*: 0.004
	*p* < 0.0001	*p* = 0.643
fit_ss_total_ave_met	*r*: 0.056	*r*: 0.019
	*p* < 0.0001	*p* = 0.063

#### 3.1.1 Sedentary time in minutes

The examination of sedentary time concerning ADHD diagnosis revealed interesting findings. Specifically, various aspects of sedentary time, including nighttime non-sleep (*r* = −0.041), daily total (*r* = −0.081), and weekly average (*r* = −0.038), demonstrated negative correlations with ADHD diagnosis. These results suggest that individuals with reduced overall sedentary time may exhibit a higher likelihood of an ADHD+ diagnosis. In contrast, daytime sedentary time exhibited a positive correlation (*r* = 0.070) with ADHD diagnosis. Importantly, these correlations retained significance even after controlling for demographics except for the weekly average. However, when controlling for demographics, the coefficient values exhibited a slight decrease, suggesting that the control variables introduced a confounding influence on the observed relationship.

#### 3.1.2 Resting heart rate

The analysis of RHR in relation to ADHD diagnosis unveiled noteworthy outcomes. Notably, RHR measurements, both overall (*r* = 0.122) and during the day (*r* = 0.119), exhibited a substantial positive correlation with ADHD diagnosis. This implies that individuals with higher RHRs are more likely to receive an ADHD+ diagnosis. These correlations remained statistically significant even after controlling for demographics. The results were also significant in within-participants analyses (overall: *r* = 0.032; during day: *r* = 0.03). These findings emphasize the potential role of RHR as a significant marker associated with ADHD diagnosis.

#### 3.1.3 Energy expenditure

Higher energy expenditure while at rest, both for daily (*r* = 0.041) and weekly average (*r* = 0.058), along with the overall total energy expenditures (*r* = 0.056), displayed positive correlations with ADHD diagnosis. These correlations indicate that individuals with higher energy expenditures while at rest, are more likely to receive an ADHD+ diagnosis. These correlations were not statistically significant after controlling for demographics. However, the fact that controlling for these variables led to moderately lower coefficient values suggested that they exerted a noticeable influence on the relationship between ADHD diagnosis and energy expenditure.

### 3.2 Multivariable logistic regression modeling

In the multivariable logistic regression analysis, the predictors were Fitbit measurements and time (date) variable and the binary outcomes were ADHD diagnosis. The model's results based on scaling the predictors are given in Table 4. We observed that six out of nine coefficient estimates were statistically significant, indicating the impact of these independent variables on the ADHD diagnosis. Despite the modest magnitudes of the coefficient values, our findings suggest a meaningful association between most of the Fitbit measurements and the ADHD diagnosis. The *z*-scores further support that these associations are unlikely to be the result of chance. We also conducted a multiple logistic regression model analysis with PCA for the overlapping variables: Group 1, consisting of fit_ss_fitbit_rest_hr, fit_ss_fitbit_restingheartrate, fit_ss_dayt_ave_met_value, and fit_ss_total_ave_met, and Group 2, consisting of fit_ss_wk_avg_sedentary_min, fit_ss_total_sedentary_min, fit_ss_fitbit_sedentarymin, and fit_ss_dayt_sedentary_min. PCA revealed that two principal components from Group 1 and one principal component from Group 2 were statistically significant. However, in both cases, the effect sizes were very small. The detailed results are shown in the Supplementary Tables S5 and S7. Additionally, a mixed-effects model was trained; however, no significant results were obtained through mixed-effect regression analysis. The lack of significant findings may be attributed to the high within-group variability observed. The Fitbit-derived measures, including daily physical activity, sedentary time, and heart rate, exhibit day-to-day fluctuations within each participant. Furthermore, measurement noise at the individual level may amplify this variability, posing a challenge for the mixed-effects model in detecting significant effects. The comprehensive results are provided in the Supplementary Table S6.

**Table 4:** Multivariable logistic regression model summary (with scaling).

Variable	Coef	Std err	*Z*-Score	*P* > |*Z*|
Intercept	−0.05778	0.02028	−2.850	0.004376
Time	−0.10591	0.02155	−4.914	8.94e-07
fit_ss_total_sedentary_min	−0.13737	0.06533	−2.103	0.035482
fit_ss_fitbit_sedentarymin	0.16524	0.04044	4.086	4.39e-05
fit_ss_dayt_sedentary_min	0.09526	0.05650	1.686	0.091804
fit_ss_wk_avg_sedentary_min	0.04500	0.03690	1.220	0.222554
fit_ss_fitbit_rest_hr	0.31254	0.10298	3.035	0.002406
fit_ss_fitbit_restingheartrate	−0.05659	0.10298	−0.549	0.582675
fit_ss_wk_average_met_value	0.14632	0.03873	3.779	0.000158
fit_ss_dayt_ave_met_value	0.23645	0.08517	2.776	0.005497
fit_ss_total_ave_met	−0.18362	0.08236	−2.230	0.025778

#### 3.2.1 Sedentary time in minutes

An increase in fit_ss_total_sedentary_min is associated with a decrease in the log-odds of ADHD diagnosis by 0.13737 units (*z*-score: −2.103, *p* = 0.0355). Conversely, an increase in fit_ss_fitbit_sedentarymin corresponds to a rise in log-odds by 0.16524 units (*z*-score: 4.086, *p* < 0.0001). Additionally, fit_ss_dayt_sedentary_min contributes to a log-odds increase of 0.09526 units, although this association is marginally significant (*z*-score: 1.686, *p* = 0.0918). Furthermore, fit_ss_wk_avg_sedentary_min does not show a significant association with ADHD diagnosis (*z*-score: 1.220, *p* = 0.2226).

#### 3.2.2 Resting heart rate

An increase in fit_ss_fitbit_rest_hr results in a rise of 0.31254 units in log-odds (*z*-score: 3.035, *p* = 0.0024). Conversely, an increase in fit_ss_fitbit_restingheartrate does not show a significant association with ADHD diagnosis (*z*-score: −0.549, *p* = 0.5827).

#### 3.2.3 Energy expenditure

An increase in fit_ss_wk_average_met_value corresponds to a log-odds rise of 0.14632 units (*z*-score: 3.779, *p* = 0.0002). Similarly, an increase in fit_ss_dayt_ave_met_value is associated with a log-odds rise of 0.23645 units (*z*-score: 2.776, *p* = 0.0055). Conversely, each unit increase in fit_ss_total_ave_met results in a decrease of 0.18362 units in log-odds (*z*-score: −2.230, *p* = 0.0258).

The variables which had statistically significant associations in our multivariable logistic regression modeling between specific Fitbit measurements and ADHD diagnosis, were selected for the machine learning classification.

### 3.3 Classification and performance

**Figure 3:**
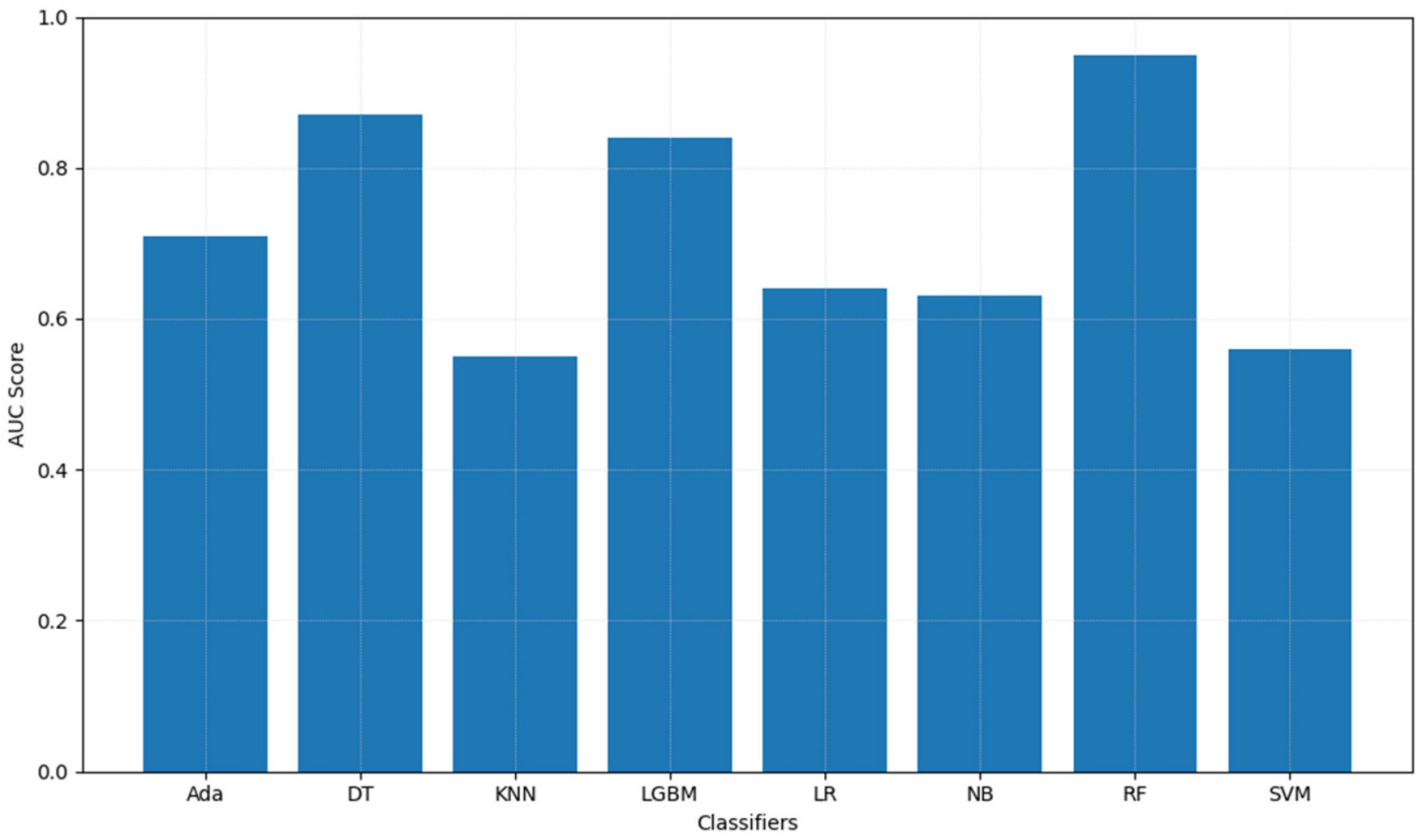
Area under the curve (AUC) scores of machine learning classifiers for distinguishing ADHD+ and ADHD− groups. This bar chart displays the AUC scores for various machine learning classifiers, highlighting their performance in distinguishing between ADHD+ and ADHD− groups. The *x*-axis represents the classifiers, and the *y*-axis shows the corresponding AUC scores.

Table 5 summarizes machine learning classifier performance in predicting ADHD diagnosis using 10-fold CV with training data and on test dataset respectively. RF outperformed other classifiers with 89.24% CV accuracy, 87.85% test accuracy, highest precision, recall, and F1-score, making it superior in ADHD diagnosis. In contrast, KNN underperformed with 53.22% CV accuracy and 53.36% test accuracy, indicating difficulty in distinguishing ADHD cases. Furthermore, strong AUC scores for RF ensemble methods indicated robust pattern learning from Fitbit data for ADHD prediction. Figure 3 illustrates classifiers' AUC scores.

**Table 5:** Classification performance of ADHD diagnosis models using Fitbit measurements.

Model	Accuracy		Precision		Recall		F1-score		AUC
	CV	Test	CV	Test	CV	Test	CV	Test	
Ada	0.6523	0.6570	0.6410	0.6477	0.6463	0.6431	0.6437	0.6454	0.71
DT	0.8846	0.8761	0.8949	0.8929	0.8639	0.8462	0.8792	0.8689	0.87
KNN	0.5322	0.5336	0.5187	0.5201	0.5148	0.5046	0.5167	0.5122	0.55
LGBM	0.7603	0.7407	0.7377	0.7187	0.7865	0.7651	0.7613	0.7412	0.84
LR	0.6138	0.6103	0.6088	0.6032	0.5738	0.5754	0.5908	0.5890	0.64
NB	0.6069	0.5893	0.5910	0.5737	0.6197	0.5990	0.6050	0.5861	0.63
RF	0.8924	0.8785	0.8813	0.8673	0.8996	0.8851	0.8904	0.8761	0.95
SVM	0.5445	0.5386	0.5727	0.5553	0.2461	0.2472	0.3443	0.3421	0.56

This table presents the performance metrics of various machine learning models for classifying ADHD diagnosis based on Fitbit measurements. The metrics include accuracy, precision, recall, F1-score, and area under the curve (AUC) for both cross-validation (CV) and test set data.

**Figure 4:**
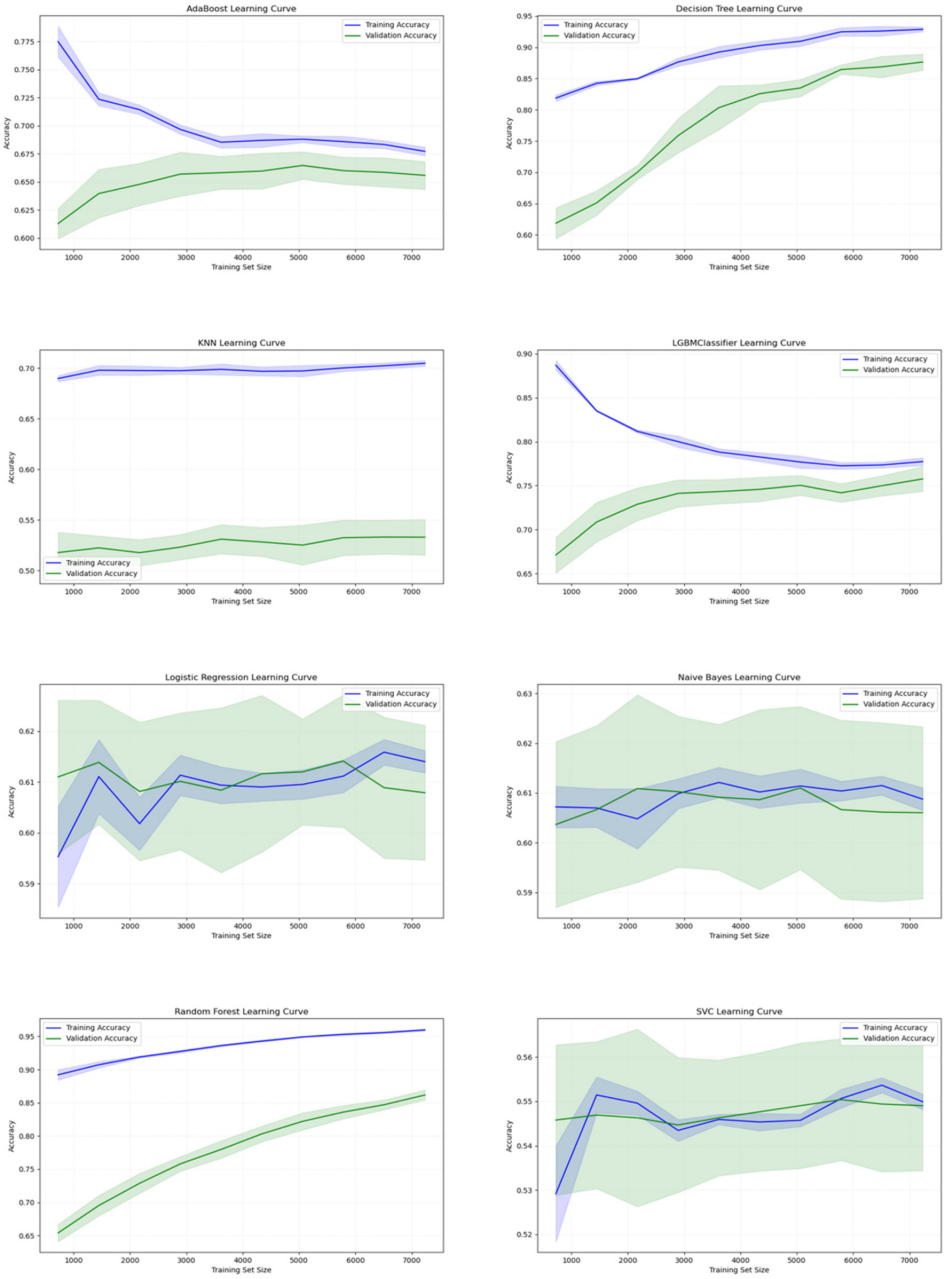
Learning curves of different machine learning classifiers using 10-fold CV with training data: training and validation accuracy across varying sample sizes. These graphs show the performance for various classifiers, with the *x*-axis representing the accuracy and the *y*-axis representing the training set size. The blue line indicates the training accuracy, while the green line represents the validation accuracy, illustrating how the model performance evolves with increasing sample sizes.

Figure 4 depicts learning curves, illustrating training and validation performance across various classification algorithms using 10-fold cross-validation, with models trained on 80% of the dataset split for training. AdaBoost, while stable, displayed limited improvement, suggesting potential undergeneralization. In contrast, DT exhibited high initial accuracy, showcasing strong generalization and consistent improvement. KNN showed potential overfitting but reasonable generalization. LGBM learning curve initially overfitted, later generalized, stabilizing with balanced accuracy. LR demonstrated limited generalization, with minor accuracy gains. NB resembled LR in limited improvement. RF initially displayed overfitting, with subsequent improvement in validation accuracy, indicating a gradually improving generalization of the model over time. SVM displayed a pattern similar to that of LR. DT and RF are favored models for accurate ADHD prediction, while SVM may require further refinement to enhance performance.

**Figure 5:**
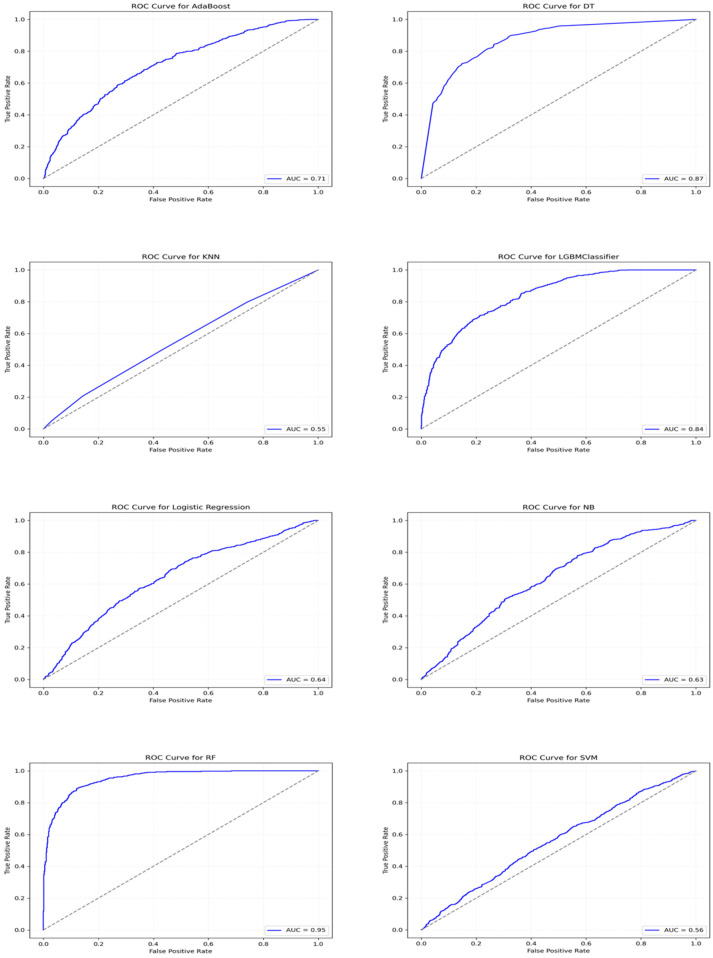
ROC curves for different classifiers: true positive rate (TPR) vs. false positive rate (FPR). These graphs display the ROC curves for various classifiers, where the *x*-axis represents the FPR, and the *y*-axis represents the TPR. The blue lines show corresponding AUC scores at different FPR and TPR values. The dotted straight line represents the diagonal line connecting the lowest (0,0) and highest (1,1) FPR and TPR ratios, which serves as a reference.

**Figure 6:**
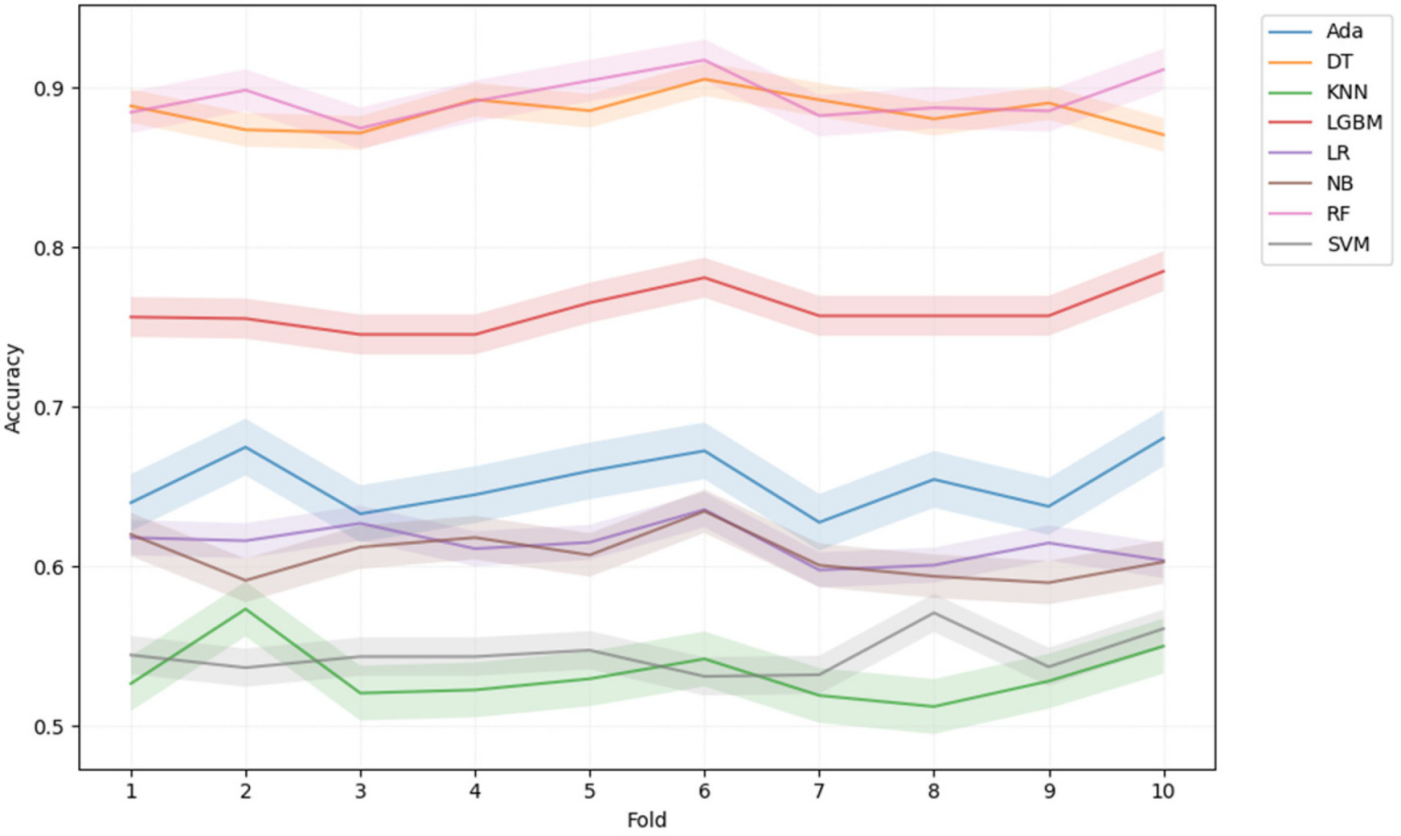
10-fold cross-validation (CV) scores for different classifiers. This graph shows the accuracy scores of various machine learning classifiers during 10-fold CV for predicting ADHD+ and ADHD− groups. The *x*-axis represents the 10 folds of the cross-validation (from 1 to 10), while the *y*-axis shows the accuracy achieved by each classifier across these folds. Different colors correspond to different classifiers, as indicated in the legend. The plot highlights the performance stability and variability of each classifier across the folds.

In Figure 5, ROC curves illustrate the classifiers' discriminative ability to distinguish ADHD+ and ADHD− cases. DT and LGBM stabilized gradually, whereas RF showed a rapid ascent and maintained high performance. In contrast, SVM, LR, and NB perform slightly better than random guessing. Figure 6 indicates that RF, DT, and LGBM are the most promising models, showing stable performance with minimal variation across cross-validation folds.

## 4 Discussion

The correlation analysis revealed statistically significant relationships between ADHD diagnosis and various Fitbit measurements, although the effect sizes were generally small in magnitude. These findings indicate that there is a statistical link between physical activity measures and ADHD. Although the observed effect sizes were small, they are clinically important when considered in conjunction with multiple factors and other diagnostic measures. Previous studies indicate that even modest associations can substantially influence clinical practice and inform the development of targeted interventions for ADHD management (39, 40). To have a clear implication of these findings, a clinical study of these associations should be explored. It is important to consider that the presence of mixed results in within-participants correlation analysis suggests variability in the relationships between variables across participants, potentially indicating heterogeneity, moderating factors, or complexities in the studied associations.

Regarding sedentary time, reduced overall sedentary time was associated with a higher likelihood of ADHD diagnosis, while increased daytime sedentary time showed a positive link to ADHD diagnosis. These findings highlight the importance of considering the timing of sedentary behavior when examining its relationship with ADHD. Our findings regarding increased daytime sedentary time align with previous research indicating a positive association between ADHD and sedentary behaviors (41–43). In contrast, our results for overall sedentary time differ from the earlier studies, which may be attributed to differences in age groups and sample sizes. However, it's important to note that our study focused on the duration of sedentary time rather than specific sedentary activities. These disparities highlight the need for further investigation to gain a more comprehensive understanding of this pattern.

Our results indicated that individuals with higher RHR are more likely to have an ADHD. These results remained true even after controlling for demographics, suggesting that RHR could serve as a strong marker for ADHD diagnosis. Our findings are consistent with several prior studies that have reported a positive association between heart rate or RHR and ADHD in both children and adults when compared to those without ADHD (30–32). Further research is needed to explore the underlying mechanisms of this relationship and its clinical implications.

Our analysis also revealed that individuals with higher energy expenditures while at rest may have an elevated likelihood of being diagnosed with ADHD. Importantly, these findings remained significant after controlling for demographics, with moderately lower coefficient values. Reduced coefficient values indicated that demographics had an impact on the association between ADHD and energy expenditure. These findings are consistent with a prior study involving a different age group and smaller sample sizes (34), which also reported a positive association between energy expenditures and ADHD. However, our results differ from another study (35), which found no significant relationship between energy expenditure and ADHD. This discrepancy may be attributed to several factors, including differences in study design, such as variations in the age and demographic characteristics of the samples, as well as variations in the methodologies used to assess Fitbit-derived measurements. These emphasize the necessity for further investigation to elucidate this relationship across different age groups, race, gender, etc.

Additionally, the Fitbit measurements variability plots (Figure 2), descriptive statistics and the Fligner-Killeen test (Supplementary Tables S7 and S9) illustrated distinct patterns, particularly characterized by increased variability in RHR and sedentary time among individuals diagnosed with ADHD. This variability suggests potential heterogeneity within the ADHD+ group, indicating that ADHD may manifest differently in individuals in terms of their physiological responses to daily activities.

This variability is particularly important, as it may reflect factors (e.g., varying levels of physical activities). Additionally, the presence of such variability could impact the results of the study by introducing extra noise, which may hinder the ability to identify consistent relationships between Fitbit-derived measures and the ADHD diagnosis in a linear model.

The multivariable logistic regression analysis identified statistically significant associations between few specific Fitbit measurements, temporal factors, and binary ADHD diagnosis. Despite modest coefficients, sedentary time, resting heart rate, and energy expenditure emerged as influential factors. Notably, sedentary time showed nuanced associations with ADHD diagnosis. The temporal variable (time/date) played a significant role. Principal component analysis highlighted the importance of certain overlapping variables in predicting ADHD outcomes. Although the mixed-effects model did not show significant results (Supplementary Table S6), the findings emphasized the potential utility of Fitbit measurements and temporal considerations in understanding and predicting ADHD diagnoses.

Among all the classifiers trained in our study, RF consistently outperformed the other classifiers across accuracy, precision, recall, F1-score, and AUC. These results underscore the robustness and dependability of the RF model in accurately distinguishing between the ADHD+ and ADHD− groups. Notably, our top-performing classifier surpassed the performance of a previous study that used a similar sample from the ABCD study, integrating multiple measures of Resting-State Functional Magnetic Resonance Imaging (rsfMRI) in adolescent brains, achieving an accuracy of 0.6916 and an AUC of 0.7408 (21) based on multiple kernel learning algorithms. Furthermore, our classifier outperformed another study conducted on a separate sample of 240 children, which utilized the temporal variability of dynamic functional connectivity from MRI brain images, achieving an accuracy of 0.78 and an AUC score of 0.84 (44) based on diagnostic model SVM. Both studies relied on expensive brain imaging methods and lab setups. In another study, Slobodin et al. achieved an accuracy of 0.87 using CPT data based on 458 children (13), employing RF and Neural Network models. However, even their best classifier fell short of our top-performing classifier in terms of accuracy, precision, and recall scores. Our classifier also outperformed an SVM classifier trained by Das et al. (14), which achieved an accuracy of 0.762 and an AUC score of 0.85 using pupillometric biomarkers and time series data.

In our analysis, KNN demonstrated the weakest performance. This suggests that KNN may not have effectively generalized the dataset, leading to difficulties in distinguishing between ADHD+ and ADHD− cases. We also observed high AUC scores for LGBM, DT and RF ensemble methods, indicating their effective learning of underlying data patterns. This highlights their suitability for predicting ADHD based on participants' daily and weekly physical activity summaries collected through Fitbit.

Based on learning curve analysis, it could be inferred that DT and RF models demonstrated strong generalization, while KNN and SVM exhibited limitations in capturing complex data patterns. ROC curve analysis further confirmed the discriminative power of these classifiers, with DT, RF, and LGBM achieving high AUC scores, indicating their proficiency in distinguishing between ADHD+ and ADHD− cases. On the other hand, KNN had a lower AUC score, suggesting its challenges in effectively classifying the two groups.

The superior performance of RF can be due to its ensemble learning approach, which combines predictions from multiple decision trees, enhancing its robustness to noise and outliers that may be present in Fitbit data (45). Additionally, RF effectively captures complex feature interactions within a low-dimensional dataset without requiring extensive feature engineering, making it particularly well-suited for our study (46).

In contrast, KNN is highly sensitive to the choice of distance metrics (47), which can be problematic when dealing with continuous and heterogeneous features, such as Fitbit measurements and demographic variables. This sensitivity may limit KNN's effectiveness in accurately distinguishing between classes in our study.

Similarly, the performance of SVM relies heavily on kernel selection (48) and optimized hyperparameter tuning (49), making it less adaptable to datasets with intricate, non-linear relationships. Given the characteristics of Fitbit data, SVM may struggle to establish meaningful decision boundaries without extensive optimization.

Our future work will explore tailored preprocessing techniques to improve the performance of KNN and SVM. For instance, incorporating polynomial feature transformations or advanced kernel functions, along with rigorous hyperparameter tuning, may enhance SVM's ability to model non-linear patterns. Likewise, optimizing distance metrics and applying appropriate feature scaling strategies could improve KNN's classification accuracy.

Overall, our machine learning experiments highlight the effectiveness of ensemble methods, particularly RF, in accurately predicting ADHD diagnosis using participants physical activity summaries collected through Fitbit. These findings provide valuable insights into the choice of classifier for ADHD classification using Fitbit measurements, such as sedentary time, RHR and energy expenditure while in rest and suggest avenues for further research and model refinement in this clinical context.

The findings suggest that there is a potentail benefits of integrating Fitbit-based measures into the screening process for ADHD. Implementing such screening tool offers a practical solution to longstanding challenges in ADHD assessment, including reducing wait times for screening and alleviating the stigma that often prevents adults from seeking evaluation—an issue compounded by the limited availability of screening protocols for adults. Adopting wearable technologies such as Fitbits and smartwatches as a non-invasive could provide a continuous monitoring tool capable of delivering objective data for both initial screening and ongoing observation.

However, the implementation of this technology in clinical settings presents several challenges. Successful integration requires comprehensive training for healthcare professionals to ensure effective use, along with managing the financial costs associated with device adoption. Moreover, maintaining consistent and reliable data collection is essential to ensure the accuracy and usefulness of the data. Patient engagement is also crucial, as their willingness to adopt these devices and share the data for clinical purposes may present a barrier.

Despite these challenges, advancements in technology offer promising solutions to facilitate integration. Through methods such as application programming interfaces (APIs) or direct data uploads, wearable devices can be easlily incorporated into clinical workflows, supporting both ADHD screening and continuous patient monitoring. This capability positions Fitbit-based measures as a valuable tool for enhancing ADHD care across diverse patient populations.

## 5 Limitation and future work

This study is based on data obtained from the ABCD study, which may not capture the entire spectrum of children and adolescents across various age groups. Additionally, the ABCD cohort is specific to certain demographic and geographic characteristics, which could introduce biases that limit the generalizability of the findings to more diverse populations. To address these limitations, future research could involve larger, more diverse Fitbit measurements to assess the applicability of these findings to broader populations. In this study, we focused on a binary classification of ADHD diagnosis (e.g., ADHD+ and ADHD−), without examining potential ADHD subtypes. However, ADHD encompasses several subtypes, each with distinct clinical characteristics. Future analyses will explore subtype-specific differences, including inattentive, hyperactive-impulsive, and combined presentations, to offer a more detailed understanding of the disorder. The current analysis employs the ABCD study's ADHD diagnosis definition based on impairment in two domains, deviating from DSM criteria. In future investigations, we aim to broaden our scope by considering other conditions alongside the ABCD ADHD diagnosis definition to establish a DSM match. Additionally, our use of Fitbit measures in this study was confined to pre-existing data available in the ABCD dataset. Future research endeavors seek to expand our exploration by incorporating raw Fitbit data, uncovering additional variables related to ADHD treatment, sedentary time, resting heart rate (RHR), and energy expenditures. Also, the ABCD dataset uses different methodologies for measuring sedentary time vs. sleep and nighttime. Specifically, sedentary time is derived from a Fitbit-based daily summary using metabolic equivalent (MET; <1.5 METs) thresholds, while sleep and nighttime are defined via heart rate criteria. This discrepancy may introduce confounding effects, as the potential influence of sleep on sedentary behavior was not explicitly examined. Future research will incorporate heart rate–based sleep measures to better understand these interactions and refine sedentary time estimations. Another limitation is the exclusion of stimulant use as a pharmacological intervention, which will be incorporated in future analyses to provide a more comprehensive understanding of treatment impacts. Lastly, the dataset lacks detailed information on ADHD treatments received by participants. Future investigations will aim to bridge this gap and may incorporate advanced methodologies, such as deep learning, to deepen insights into ADHD characteristics and management.

## 6 Conclusion

This study illuminates the associations between Fitbit-derived physical activity summaries and ADHD diagnosis (e.g., ADHD+ and ADHD− groups) using the ABCD dataset. Our findings demonstrate that wearable technology, showed by the performance of the Random Forest classifier, holds promise in the realm of ADHD prediction and diagnostic applications. Our results provide a foundation for further exploration and the eventual integration of wearable data into the clinical landscape, fostering a deeper understanding of ADHD and advancing the accuracy of its identification.

## Data Availability

Publicly available datasets were analyzed in this study. This data can be found here: https://nda.nih.gov/study.html?id=2313.
